# Draft Genome Sequence of a Novel Iflavirus from Leafhoppers (Exitianus capicola) in Iraq

**DOI:** 10.1128/mra.00108-22

**Published:** 2022-04-18

**Authors:** Zhuang-Xin Ye, Adnan A. Lahuf, Mohammed D. Salman, Yan Zhang, Jun-Min Li

**Affiliations:** a State Key Laboratory for Managing Biotic and Chemical Threats to the Quality and Safety of Agro-products, Key Laboratory of Biotechnology in Plant Protection of Ministry of Agriculture and Zhejiang Province, Institute of Plant Virology, Ningbo University, Ningbo, China; b Plant Protection Department, Agriculture College, University of Kerbala, Karbala, Iraq; Queens College CUNY

## Abstract

The draft genome sequence of a novel virus was determined from a leafhopper insect (Exitianus capicola) via transcriptomic sequencing and rapid amplification of cDNA ends. Results of the sequence alignment suggested that the new virus is a member of the genus *Iflavirus*, family *Iflaviridae*. Thus, it was proposed to be named Exitianus capicola iflavirus 2 (ECIV2). ECIV2 has a single positive-strand RNA genome of 7,821 nucleotides [excluding the poly(A) tail] containing a single open reading frame (ORF) with typical iflavirus conserved domains, including a picornavirus capsid protein-like domain, a cricket paralysis virus capsid-like domain, a helicase, a peptidase, and an RNA-dependent RNA polymerase domain.

## ANNOUNCEMENT

The leafhopper Exitianus capicola is one of numerous species in the *Exitianus* genus and is considered among the most common tropical and temperate grassland leafhoppers (1). *Iflavirus* is the only genus belonging to the *Iflaviridae* family; all 15 species of this genus are confined exclusively to arthropods, mostly insects (2).

During the growth season of 2020, adult leafhopper samples were collected from several rice fields located in Al Diwaniyah Province (31.51N, 45.3E), Iraq. For RNA sequencing, total RNA was extracted from all samples using TRIzol reagent (Invitrogen, USA). Sequencing libraries were generated using the NEBNext Ultra RNA library preparation kit for Illumina (New England Biolabs, USA) following the manufacturer’s recommendations. Paired-end sequencing using an Illumina HiSeq 2000 platform was performed based on standard procedures followed by the BGI Company, Hongkong Tech Solution, China. Subsequently, a total of 30,698,435 paired-end raw reads (100 nucleotides [nt] long) generated were subjected to quality trimming and adaptor removal by Trimmomatic version 0.39 with default parameters. The clean data obtained were *de novo* assembled using SPAdes version 3.15.2 with default parameters. (3). The assembled contigs were compared with reference sequences of metazoan mitochondrial genomes, which confirmed that the leafhopper species was Exitianus capicola (Hemiptera: Cicadellidae), with a cytochrome *c* oxidase I (COI) sequence identical to the COI sequence deposited in GenBank with accession number MK188553.1. In addition, to identify the iflavirus-like viral contig, the assembled contigs were compared to nonredundant limited species viruses with –taxid 10239. The result indicated the existence of iflavirus-like contigs, which was verified by reverse transcription (RT)-PCR. Moreover, the draft genome sequence of this putative iflavirus was successfully obtained using rapid amplification of cDNA ends (RACE) with a SMARTer RACE amplification kit (Takara, China). The primers used for RT-PCR and RACE are listed in Table 1.

**TABLE 1:** Primers used in this study

Primer^*a*^	Sequence (5′ to 3′)	Purpose
Long primer	CTAATACGACTCACTATAGGGCAAGCAGTGGTTTTCCCAGTCACG	Amplification of 5′/3′-RACE fragment
Short primer	CTAATACGACTCACTATAGGGC	
5′-RACE GSP	CCCACTGTTGTTTAACAAAATCAATTGACA	Amplification of 5′-RACE fragment of ECIV2
3′-RACE GSP	GGCTCAATTGGACAAAAGATCTGTTTTGGA	Amplification of 3′-RACE fragment of ECIV2
ECIV1-F	ACCTTTAGAGGTTTTGATCCTGT	Amplification of ECIV1 genome
ECIV1-R	AAGCCATCATTTCGGGCTCA	
ECIV2-F	TTATAAGTCTATGTTATCAATG	Amplification of ECIV2 genome
ECIV2-R	GAGAAATAGAAATTGATTCATAC	

a GSP, gene-specific primer; F, forward; R, reverse.

**FIG 1:**
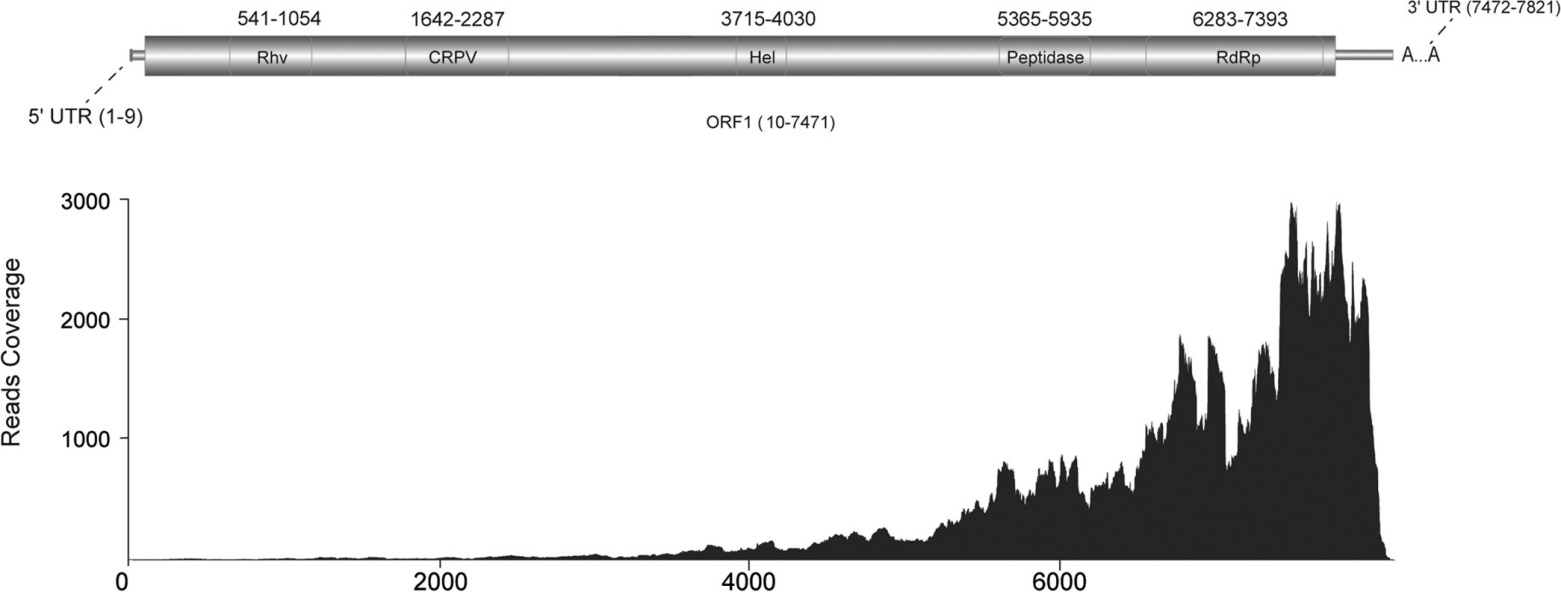
Genome organization of ECIV2 and raw read coverage of ECIV2. Hel, viral helicase domain; RdRp, RNA-dependent RNA polymerase domain.

The full draft genome of the candidate iflavirus obtained was 7,821 nt in length [ignoring the poly(A) tail], with a GC content of 34.4%. The predicted long open reading frame (ORF) of the discovered virus (Exitianus capicola iflavirus 2 [ECIV2]) was from nucleotide position 10 to nucleotide position 7471, encoding 2,487 amino acids, as predicted using the Expasy online server (https://web.expasy.org/translate), a 9-nt untranslated region (UTR) at the 5′ end, and a 349-nt UTR at the 3′ end. The prediction of conserved domains using InterProScan (https://www.ebi.ac.uk/interpro) showed that the ORF of ECIV2 includes a picornavirus capsid protein (Rhv)-like domain, a cricket paralysis virus (CrPV) capsid-like domain, a helicase domain, a peptidase domain, and an RNA-dependent RNA polymerase domain (Fig. 1). BLASTP analysis of the amino acid sequence of the virus was performed against the total nonredundant protein database. The analysis revealed that this virus has a sequence similarity of 43.18% with respect to the polyprotein of ACT flea iflavirus.

### Data availability.

The draft genome sequence of ECIV2 was deposited in the GenBank database under the accession number OL778829. The corresponding transcriptome data were deposited under the accession number SRR18046885.
